# Supplementary material for: The TBC1D31/praja2 complex controls primary ciliogenesis through PKA‐directed OFD1 ubiquitylation
Source: EMBO J. 2021 May 2;40(10):e106503. doi: 10.15252/embj.2020106503 (PMC8126939; doi:10.15252/embj.2020106503)
Supplement: Supplementary file 1 — Appendix [file EMBJ-40-e106503-s010.pdf]

APPENDIX

The TBC1D31/praja2 complex controls primary ciliogenesis through PKA-directed OFD1 ubiquitylation.

Emanuela Senatore[1]*, Francesco Chiuso[1]*, Laura Rinaldi[1], Daniela Intartaglia[2], Rossella Delle Donne[1], Emilia Pedone[3], Bruno Catalanotti[4], Luciano Pirone[3], Bianca Fiorillo[4], Federica Moraca[4], Giuliana Giamundo[2], Giovanni Scala[5], Andrea Raffeiner[6,7], Omar Torres-Quesada[6,7], Eduard Stefan[6,7], Marcel Kwiatkowski[6], Alienke van Pijkeren[6], Manuela Morleo[2], Brunella Franco[2,8], Corrado Garbi[1], Ivan Conte[2,5] and Antonio Feliciello[1#].

EMBOJ-2020-106503

Appendix figures

Appendix figure S1. Characterization of the anti-TBC1D31 polyclonal antibodies raised against residues 239-358 of human TBC1D31.

A. HEK293 cells were transiently transfected with control siRNA (siCNT) or siRNA targeting TBC1D31 (siTBC1D31). Lysates were immunoblotted with anti-TBC1D31 antibody. Where indicated, the antibody was preincubated with purified recombinant immunogen. As control, a pre-immune serum was used.

B. HEK293 cells were fixed and immunostained with anti-TBC1D31, anti-γ-tubulin and DRAQ5.

Appendix figure S2. Modeling of TBC1D31/praja2 interaction.

A. Superposition between the praja2$_{530\text{-}570}$ predicted docking pose of AutoDock Vina (yellow cartoon) and the FlexPepDock refinement (cyan cartoon) against TBC1D31 (Q941-A970) (red cartoon).

B. Plot of the energy landscape (kcal/mol) sampled by Rosetta FlexPepDock. The funnel-like shape of the docking scores suggest the global minimum as a near-native complex.

C. Superposition among the first, the second and the third most populated praja2$_{550\text{-}570}$ clusters (green, cyan and pink cartoon, respectively) with TBC1D31$_{wt}$ from ~2µs-long MD simulation.

D. Table with the percentage of cluster's population.

Appendix figure S3. Microscale thermophoresis analysis and circular dichroism of TBC1D31 peptides.

A. Microscale thermophoresis traces. panels 1, 2 and 3 correspond to titration of TBC1D31 peptide vs GST-praja2$_{530\text{-}570}$, GST-praja2$_{550\text{-}610}$, GST-praja2$_{590\text{-}630}$ respectively; panels 4, 5 and 6 show titration of TBC1D31$_{AA}$, TBC1D31$_{ADA}$ and TBC1D31$_{wt}$ vs GST-praja2$_{530\text{-}570}$ respectively. F_0 and F_1 correspond to the fluorescence of unbound state and bound state respectively.

B. Far-UV CD spectra of: TBC1D31$_{wt}$ (green line), TBC1D31$_{AA}$ (blue line) and TBC1D31$_{ADA}$ (red line).

Appendix figure S4. cAMP stimulation regulates TBC1D31 binding to OFD1.

A. HEK293 cells were co-transfected with vectors encoding for GFP-TBC1D31 and flag-OFD1 or flag-S735A, starved for 24 hours and then treated with forskolin (40 µM) for 1 hour. Lysates were immunoprecipitated with anti-flag antibody. Precipitates and an aliquot of lysates were immunoblotted with anti-GFP and anti-flag antibodies.

B. Quantitative analysis of the experiments shown in A. A mean value ± SD of three independent experiments is shown. Student's t test p **< 0,01.

Appendix figure S5. TBC1D31 and praja2/PKA/OFD1 network is involved in the correct Medaka fish development.

A. Stereo-microscopic images of wild-type, hOFD1wild-type, $hOFD1_{S735D}$, hpraja2rm, Ol-TBC1D31 KD + hpraja2rm and $hOFD1_{S735D}$ + hpraja2rm injected Medaka larvae, at stage 40.

B. Representative immunoblots of OFD1 protein from indicated Medaka embryos.

C. Representative immunoblots of OFD1 and praja2 proteins from indicated Medaka embryos.

Appendix figure S6. Inhibition of proteasome activity affects primary ciliogenesis.

A. NIH3T3 cells were serum deprived for 36 hours and then treated for 8 hours with MG132 (10 μM), a proteasomal inhibitor or Bafilomycin (1 μM), an autophagy inhibitor. Cells were fixed and stained for acetylated-tubulin and DRAQ5.

B. Statistical analysis of two independent experiments show in A that gave similar results.

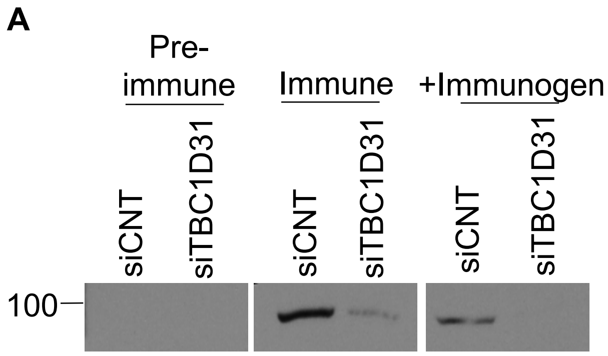

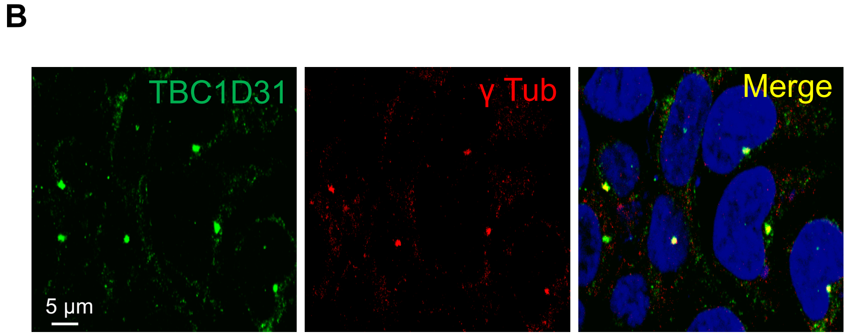

Appendix Figure S1

A

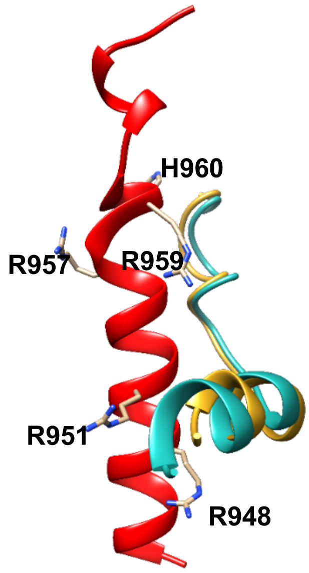

B

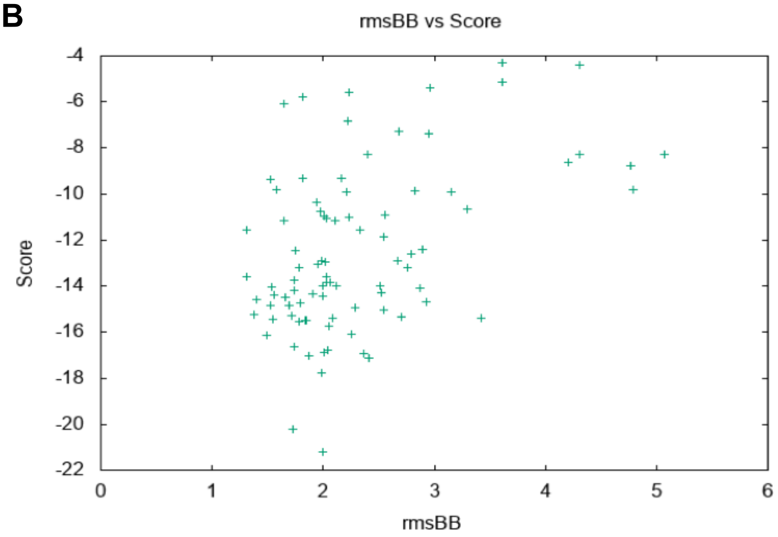

C

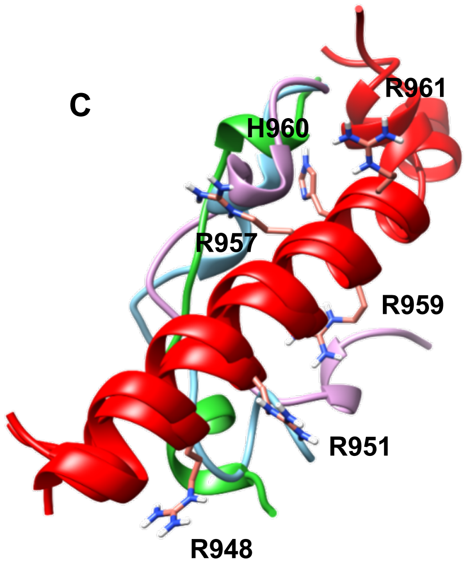

D

cluster0	58.7 %
cluster1	21.5 %
cluster2	19.3%

Appendix Figure S2

A

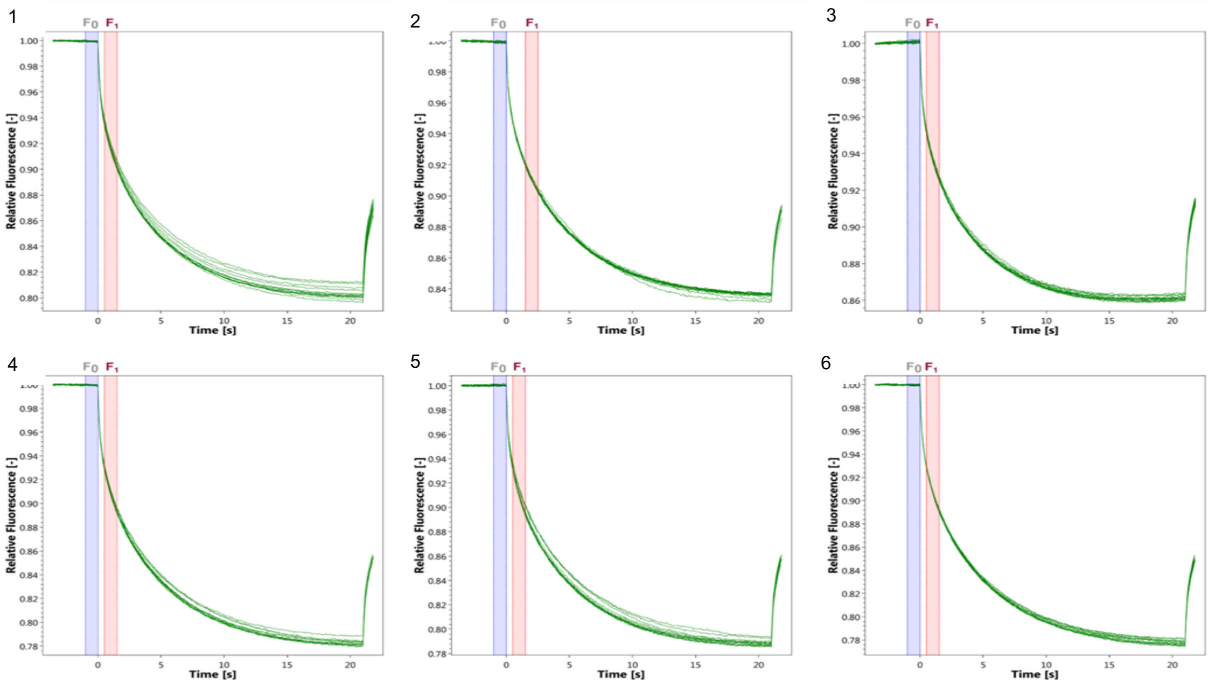

B

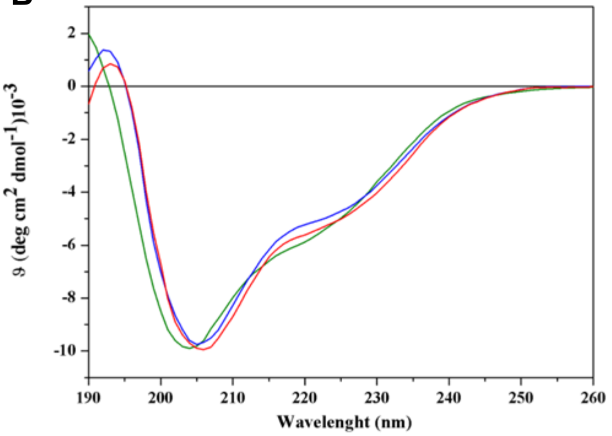

Appendix Figure S3

A

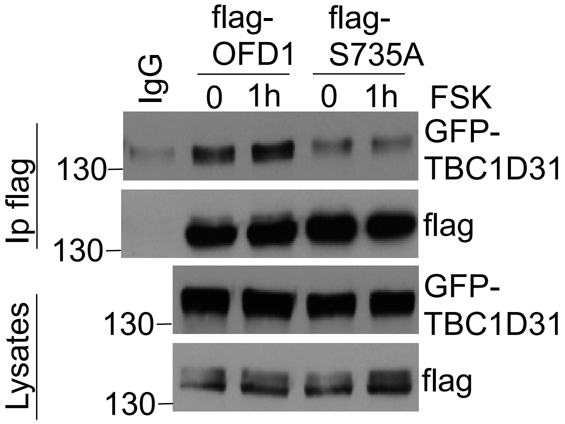

B

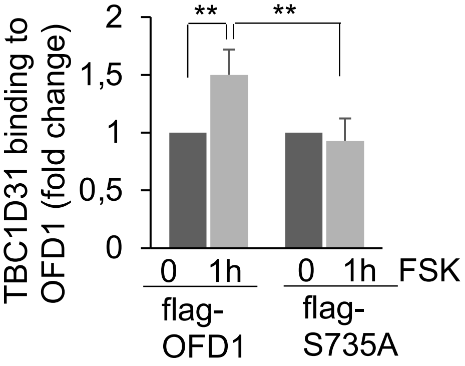

Appendix Figure S4

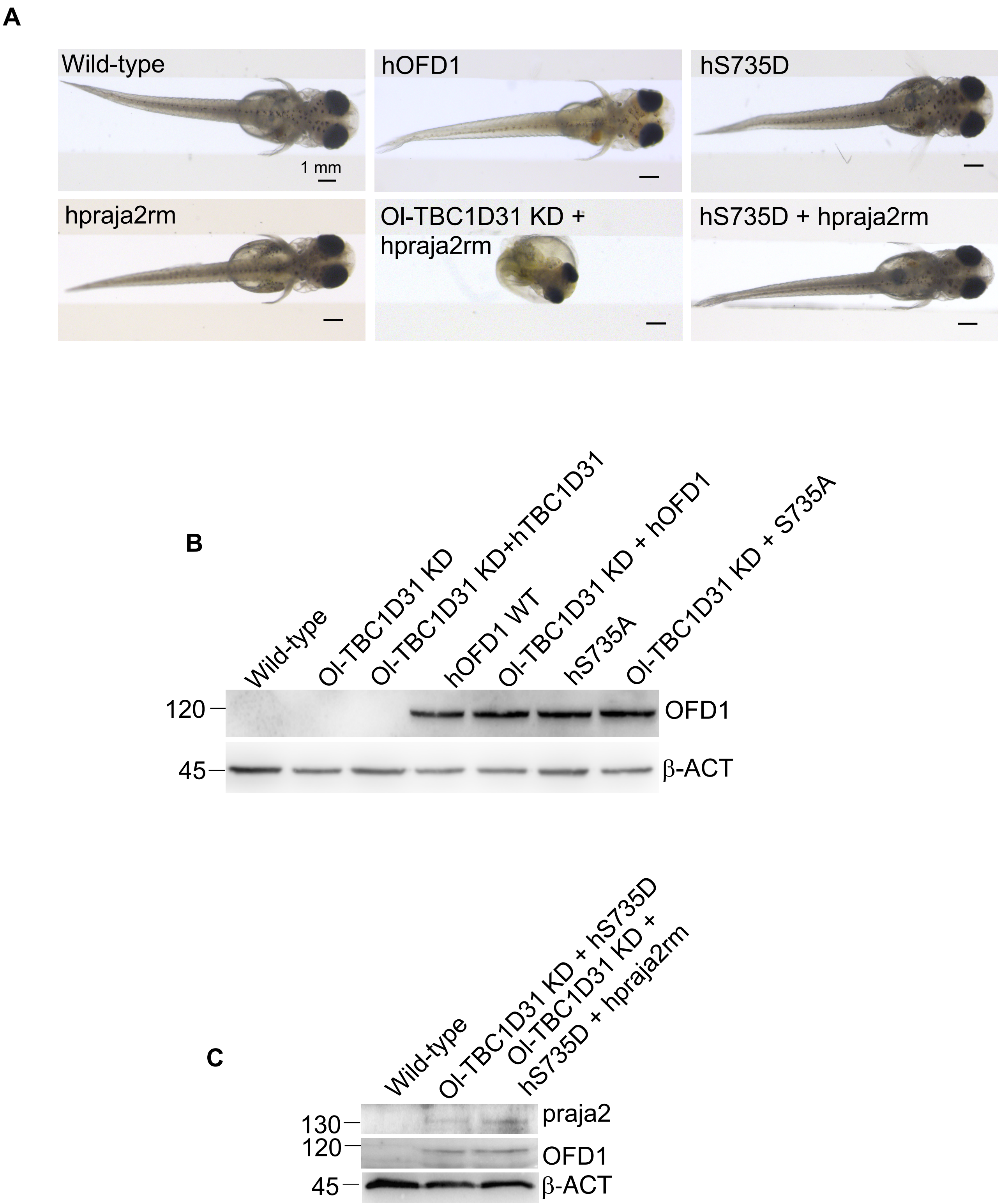

Appendix Figure S5

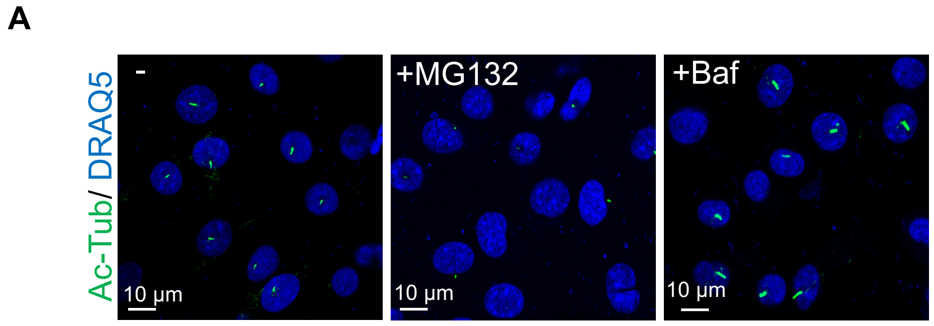

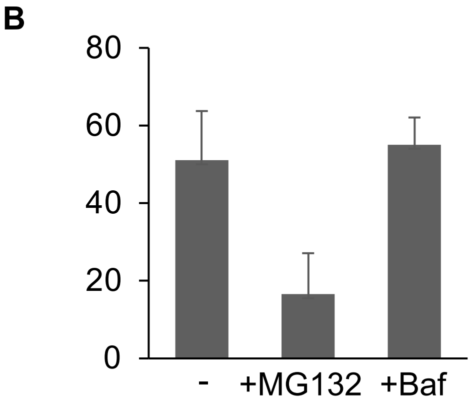

Appendix Figure S6
