# Supplementary material for: The TBC1D31/praja2 complex controls primary ciliogenesis through PKA‐directed OFD1 ubiquitylation
Source: EMBO J. 2021 May 2;40(10):e106503. doi: 10.15252/embj.2020106503 (PMC8126939; doi:10.15252/embj.2020106503)
Supplement: Supplementary file 2 — Expanded View Figures PDF [file EMBJ-40-e106503-s004.pdf]

Expanded View Figures

Figure EV1. Localization of praja2 and TBC1D31 at centrosome.

A HEK293 cells were fixed and stained with anti-praja2 and anti-γ-tubulin antibodies. Nuclei were stained with DRAQ5.
B HEK293 cells transiently transfected with control siRNA or siRNA targeting endogenous praja2 were stained with anti-praja2 antibody and DRAQ5.
C Immunoblot analysis of TBC1D31 and Hsp90 in siRNA-silenced cells.
D Cells were transiently transfected with control siRNA or siRNA targeting endogenous TBC1D31. Total RNA was extracted and analysed by quantitative RT–PCR.
E Cells transiently transfected with control siRNA or siRNA targeting endogenous praja2 were stained for TBC1D31, γ-tubulin and DRAQ5.
F Immunoblot analysis of praja2 and Hsp90 in siRNA-silenced cells.

© 2021 The Authors

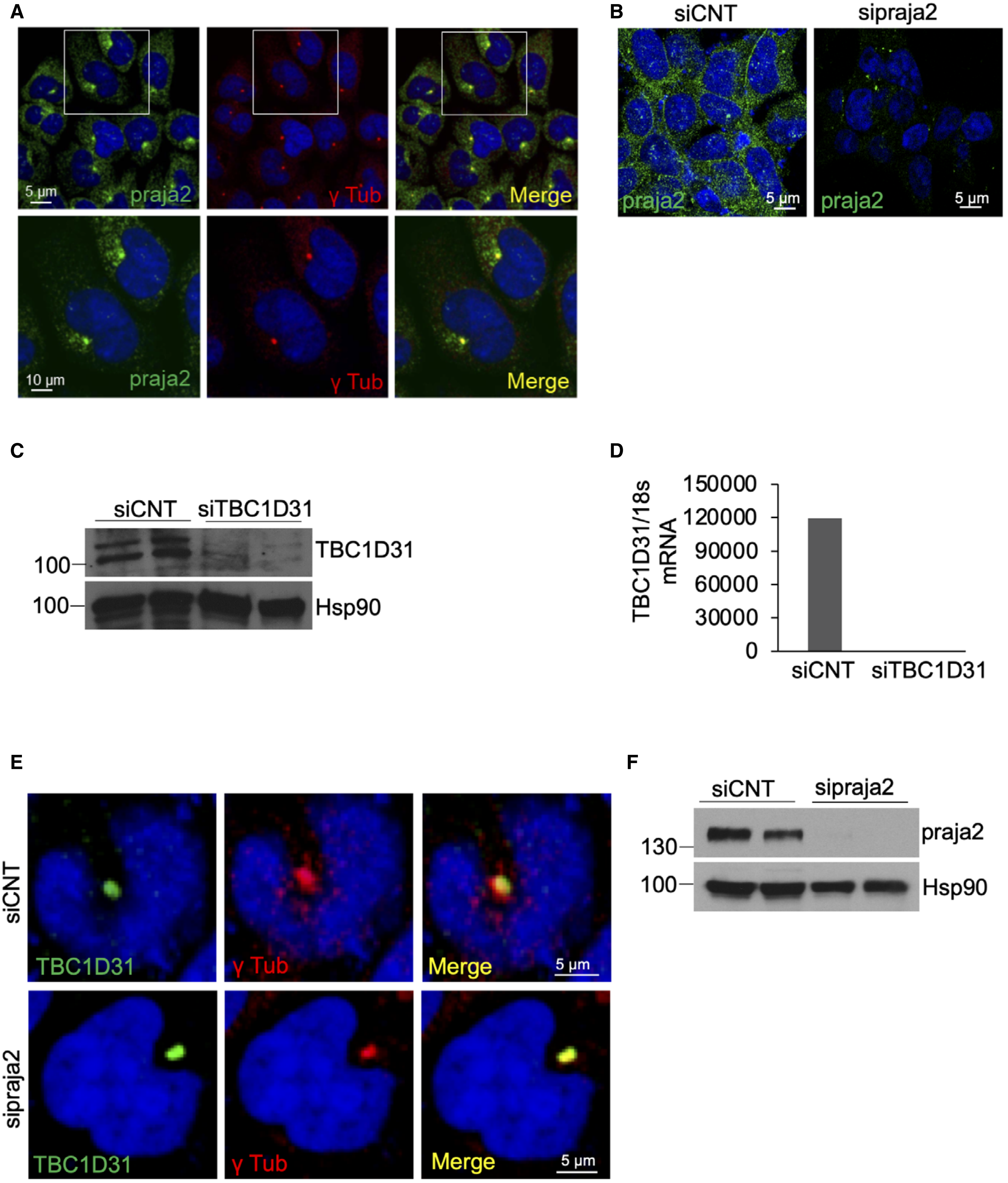

Figure EV1.

 © 2021 The Authors

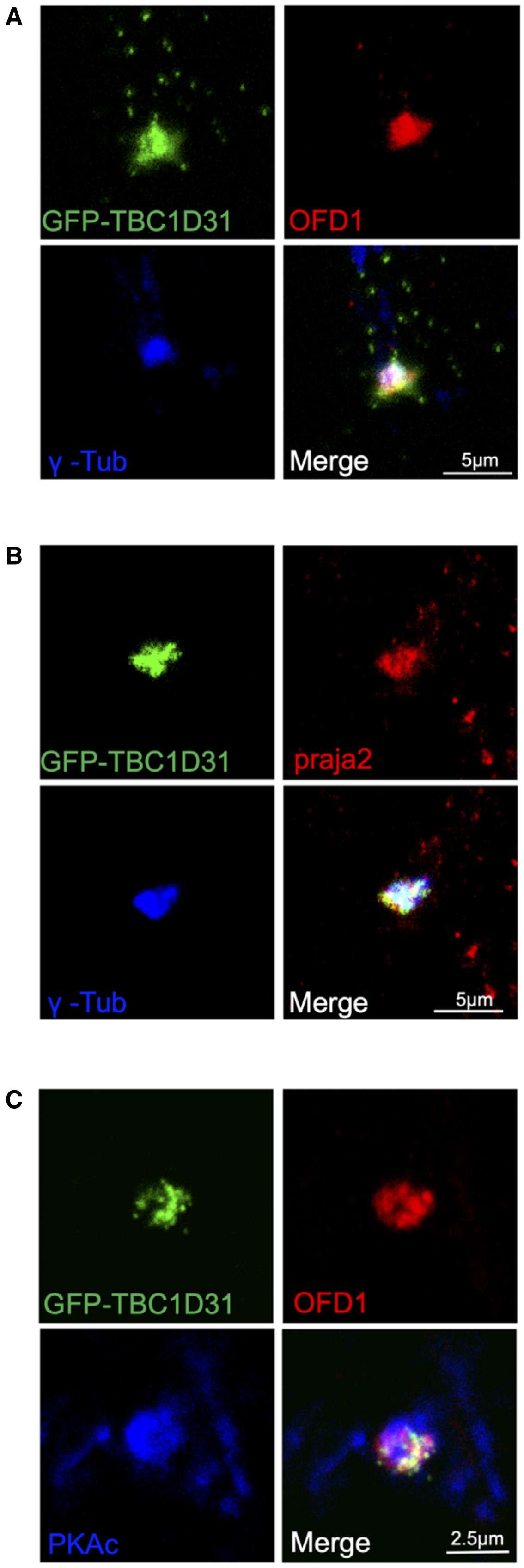

Figure EV2. Centrosomal localization of praja2, TBC1D31, OFD1 and PKAc.

A HEK293 cells transfected with GFP-TBC1D31 were fixed and immunostained with anti-OFD1 and anti-γ-tubulin antibodies.
B HEK293 cells transfected with GFP-TBC1D31 were fixed and immunostained with anti-praja2 and anti-γ-tubulin antibodies.
C HEK293 cells transfected with GFP-TBC1D31 were fixed and immunostained with anti-OFD1 and anti-PKAc antibodies.

© 2021 The Authors

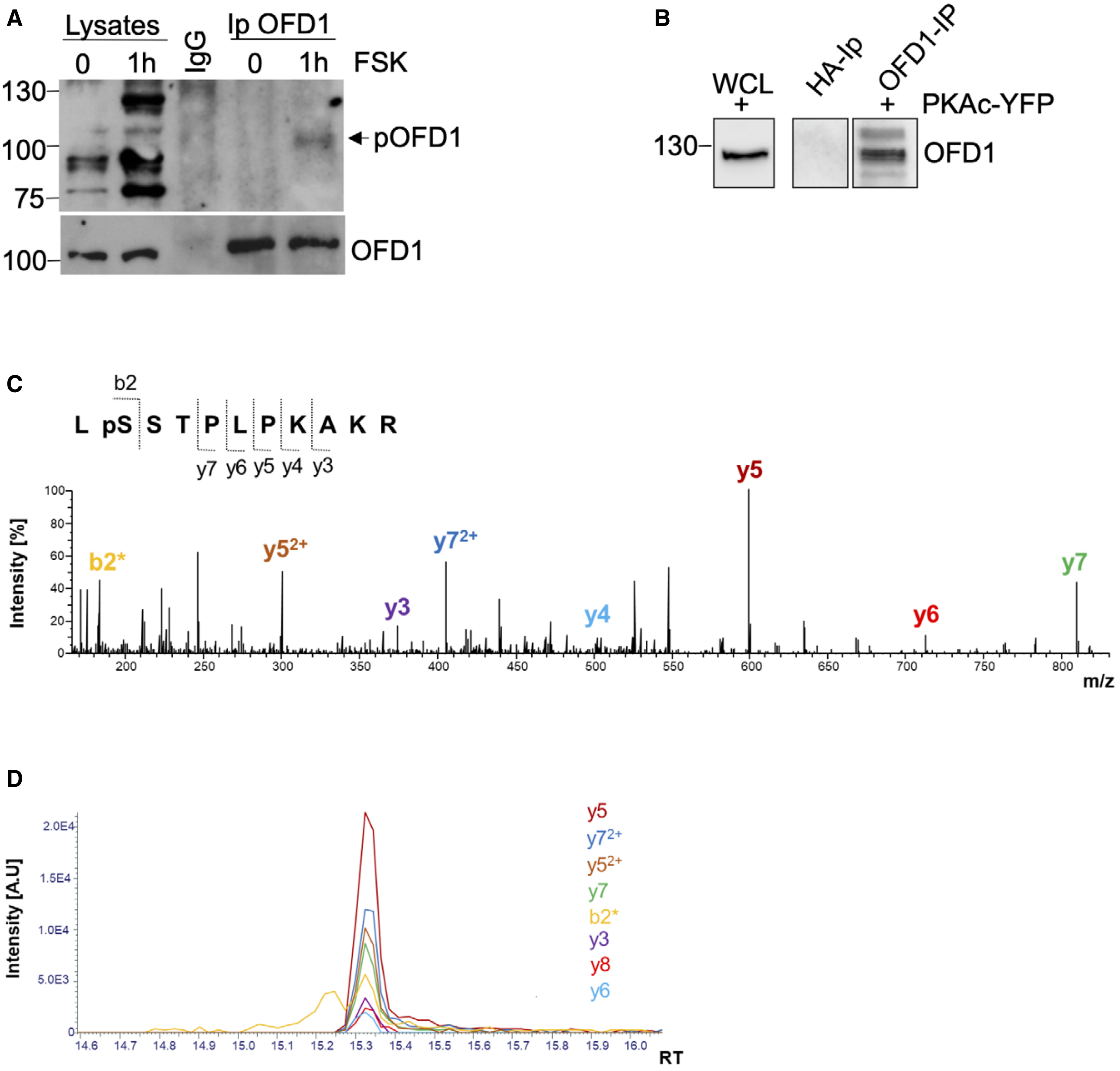

Figure EV3. Phosphorylation of endogenous OFD1.

A HEK293 cells were starved for 24 h and then treated with FSK (40 μM) for 1 h. Lysates were immunoprecipitated with anti-OFD1 antibody or with control IgG. Lysates and precipitates were immunoblotted with anti-phospho-(K/R)(K/R)X(S*/T*) and anti-OFD1 antibodies.

B Western blot analysis of affinity-isolated endogenous OFD1. Hela cells transiently expressing PKAc-YFP were serum-deprived for 48 h and lysed. Total lysates were immunoprecipitated with anti-HA (control IP) or with anti-OFD1 antibody. An aliquot of whole cell lysate (WCL) and the precipitates were immunoblotted for OFD1. Gel-isolated OFD1-containing fragments were subjected to mass spectrometric analysis.

C Fragment spectrum of *m/z* 426.5708 [M + 3H]3+ with the identified y- and b-fragment ions of the phosphorylated OFD1 peptide LpSSTPLPKAKR. b2*: b2-fragment ion containing dehydrolalanine, which represents the formerly phosphorylated serine residue. Dehydroalanine is formed from phosphoserine during collision-induced dissociation in HCD (Higher-energy Collisional Dissociation) due to the neutral loss of H3PO4. The phosphorylated peptide was identified by a Sequest data base search using Percolator with a q-value of 7e-4.

D Extracted ion chromatograms (EICs) of the b- and y-fragment ions showing that all fragment ions co-elute.

 © 2021 The Authors

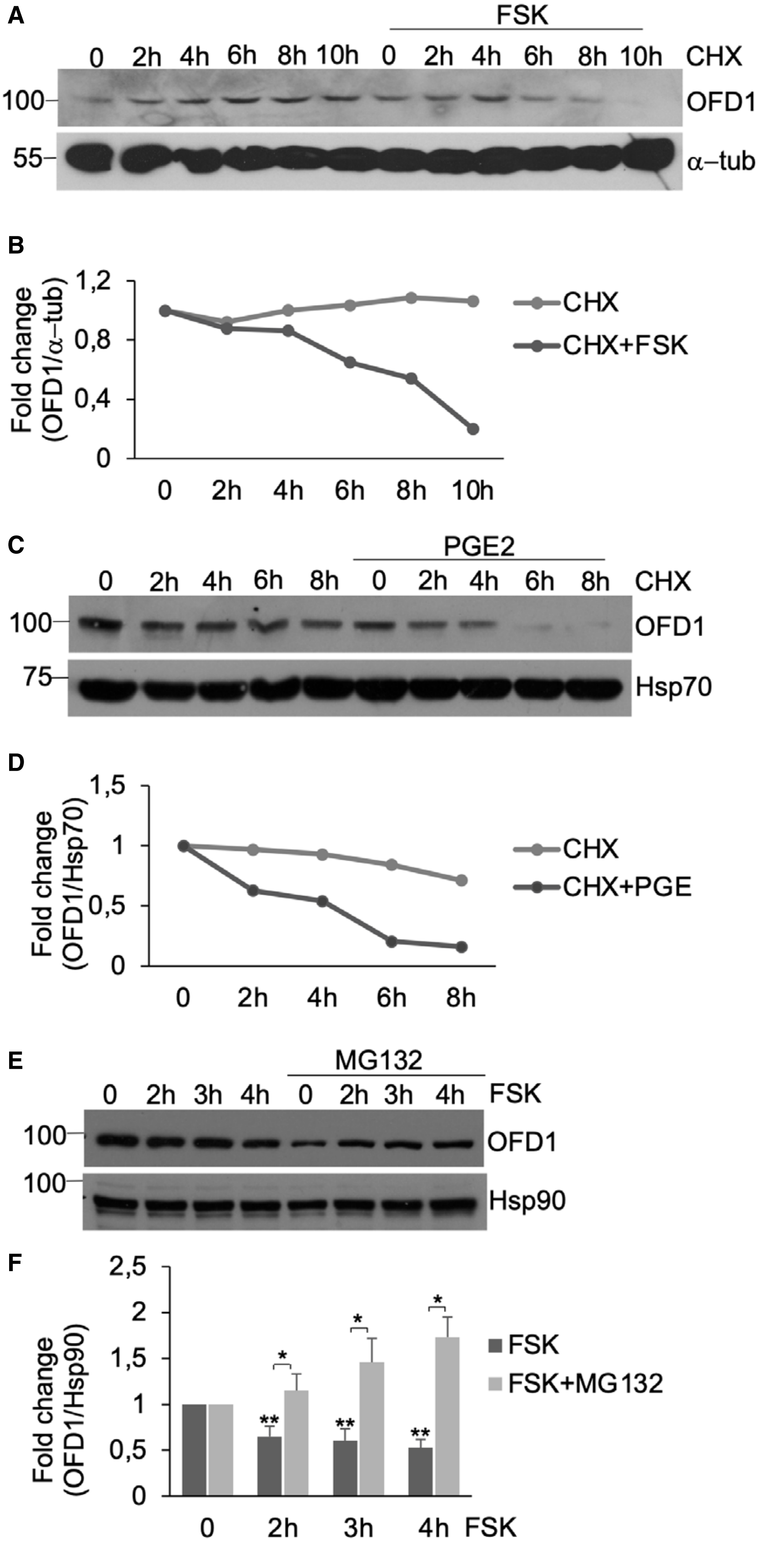

Figure EV4. cAMP stimulation regulates OFD1 stability.

A HEK293 cells were serum-deprived for 24 h, treated with cycloheximide (100 μM) with or without FSK (40 μM) and harvested at the indicated time points. Lysates were immunoblotted with anti-OFD1 and anti-α-tubulin antibodies.

B Quantitative analysis of the experiments shown in (A). A mean value of two independent experiments that gave similar results is shown.

C Same as in (A), with the exception that PGE2 (1 μM) was used instead of FSK. Lysates were immunoblotted with anti-OFD1 and anti-Hsp70 antibodies.

D Quantitative analysis of the experiments shown in (C). A mean value of two independent experiments that gave similar results is shown.

E HEK293 cells were serum-deprived for 24 h, treated with cycloheximide (100 μM) and treated with FSK (40 μM) for the indicated times. Where indicated, MG132 (20 μM) was added to the medium. Lysates were immunoblotted with anti-OFD1 and anti-Hsp90 antibodies.

F Quantitative analysis of the experiments shown in (E). A mean value ± SD of three independent experiments is shown. Student's *t* test *$P < 0.05$, **$P < 0.01$.

© 2021 The Authors

Figure EV5. Role of TBC1D31, OFD1 and FSK in ciliogenesis and cilium morphology.

A Immunostaining analysis of serum-deprived HEK293 cells for TBC1D31, acetylated α-tubulin and DRAQ5.
B Human U-87MG glioblastoma cells transfected with control siRNA (siCNT) or siRNA targeting TBC1D31 (siTBC1D31) were serum-deprived for 36 h, fixed and stained for acetylated-tubulin, TBC1D31 and DRAQ5.
C Quantitative analysis of the experiments shown in (B). A mean value ± SD of three independent experiments is shown. Student's *t* test $^{**}P < 0.01$.
D HEK293 cells were transiently transfected with flag-OFD1 or flag-S735A, serum-deprived for 36 h, fixed and immunostained for flag, acetylated-tubulin and DRAQ5.
E NIH3T3 cells transiently expressing flag-OFD1 or flag-S735A were serum-deprived for 36 h, treated with FSK (6 h), fixed and immunostained for flag, acetylated-tubulin and DRAQ5.
F Statistical analysis of the experiments shown in (E). A mean value ± SD of three independent experiments is shown. Student's *t* test, $^{***}P < 0.001$, $^{**}P < 0.01$.

 © 2021 The Authors

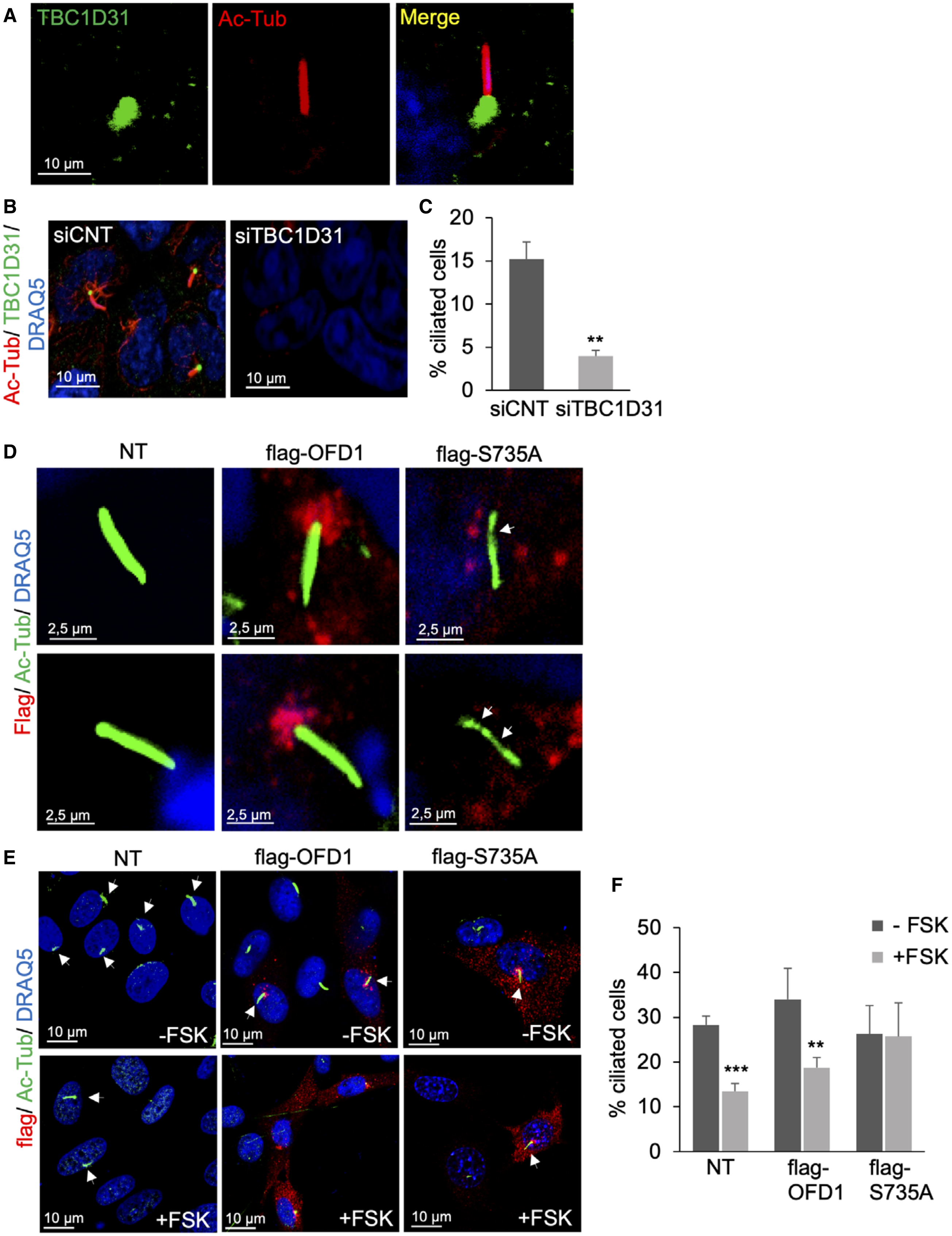
A
TBC1D31
Ac-Tub
Merge
10 µm
B
Ac-Tub/ TBC1D31/ DRAQ5
siCNT
siTBC1D31
10 µm
10 µm
C
% ciliated cells
20
15
10
5
0
**
siCNT siTBC1D31
D
NT
flag-OFD1
flag-S735A
Flag/ Ac-Tub/ DRAQ5
2,5 µm
E
NT
flag-OFD1
flag-S735A
flag/ Ac-Tub/ DRAQ5
10 µm
-FSK
+FSK
F
- FSK
+FSK
% ciliated cells
50
40
30
20
10
0
***
**
NT
flag-OFD1
flag-S735A

Figure EV5.
