# Supplementary material for: The TBC1D31/praja2 complex controls primary ciliogenesis through PKA‐directed OFD1 ubiquitylation
Source: EMBO J. 2021 May 2;40(10):e106503. doi: 10.15252/embj.2020106503 (PMC8126939; doi:10.15252/embj.2020106503)
Supplement: Supplementary file 3 — Movie EV1 [file EMBJ-40-e106503-s002.zip › EMBOJ-2020-106503_Movie_EV1_with_legend/EMBOJ-2020-106503_legend_to_movie_EV1.pdf]

Movie EV1. Movie representation of 2 microseconds long Molecular Dynamics simulations showing the proposed binding mode of praja2$_{530-570}$ (green cartoon) against TBC1D31 (red cartoon). Key interacting arginine-rich stretch R1053-R1057 of TBC1D31 and F553-D558 of Praja-2 are represented in stick colored by atom type.
