# Supplementary material for: The TBC1D31/praja2 complex controls primary ciliogenesis through PKA‐directed OFD1 ubiquitylation
Source: EMBO J. 2021 May 2;40(10):e106503. doi: 10.15252/embj.2020106503 (PMC8126939; doi:10.15252/embj.2020106503)
Supplement: Supplementary file 4 — Source Data for Expanded View [file EMBJ-40-e106503-s003.zip › EMBOJ-2020-106503_SourceDataForForExpandedView/EMBOJ-2020-106503_ourceDataForAppendix.pptx]

## Slide 1
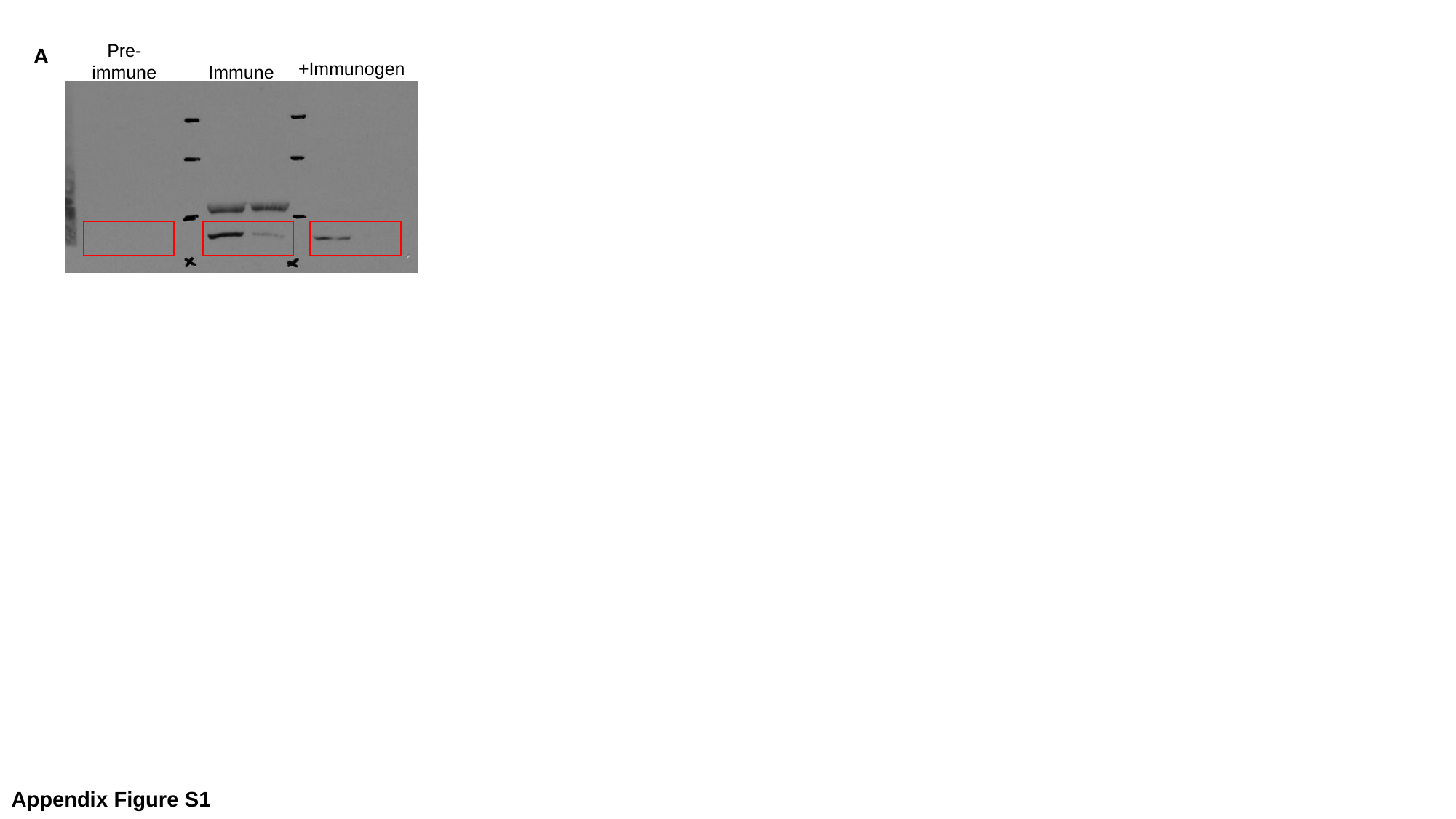

Pre-immune
+Immunogen
Immune
A
Appendix Figure S1

## Slide 2
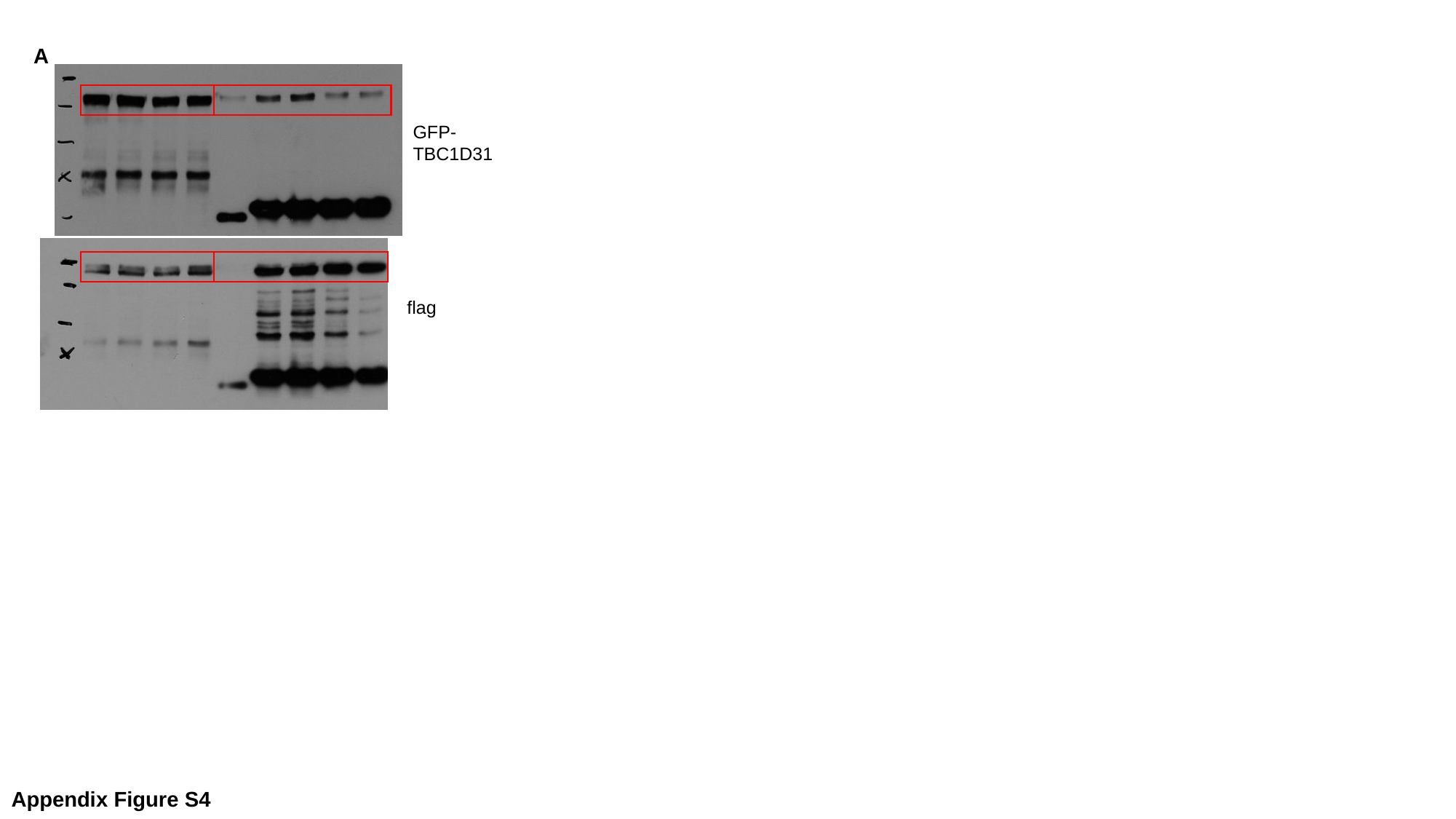

A
GFP-TBC1D31
flag
Appendix Figure S4

## Slide 3
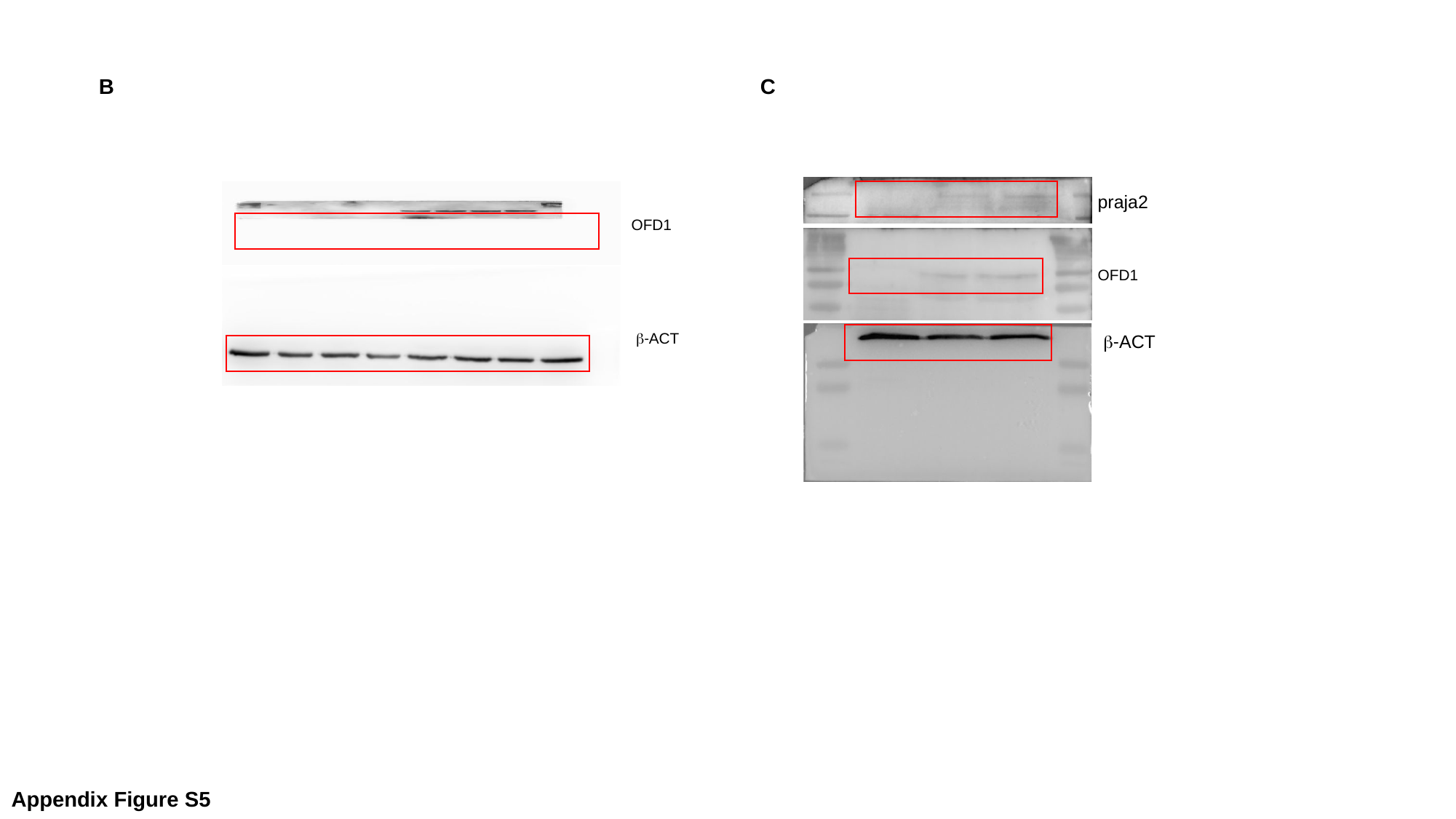

B
C
praja2
OFD1
β-ACT
OFD1
β-ACT
Appendix Figure S5
