# Supplementary figures and images for: The TBC1D31/praja2 complex controls primary ciliogenesis through PKA‐directed OFD1 ubiquitylation
Source: EMBO J. 2021 May 2;40(10):e106503. doi: 10.15252/embj.2020106503 (PMC8126939; doi:10.15252/embj.2020106503)

C

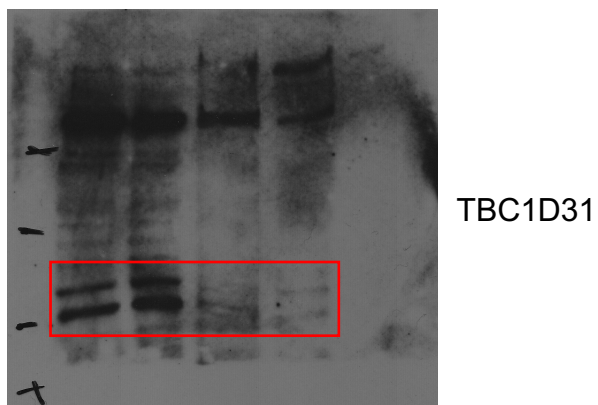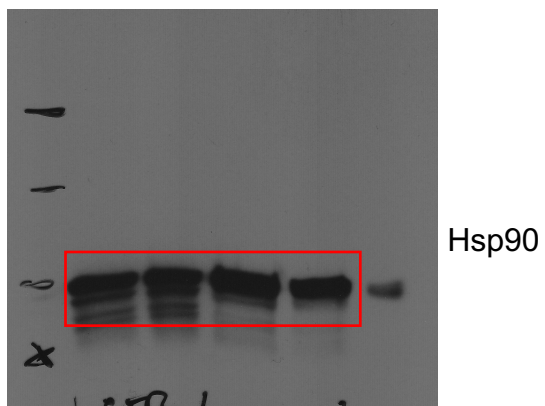

F

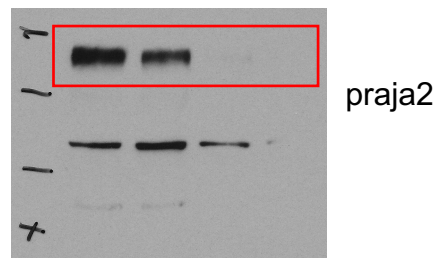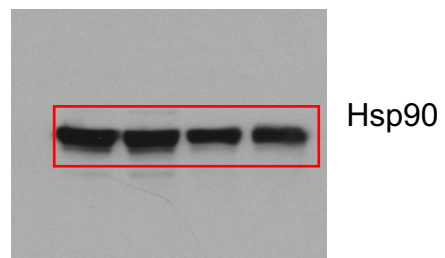

Fig. EV1

**A**

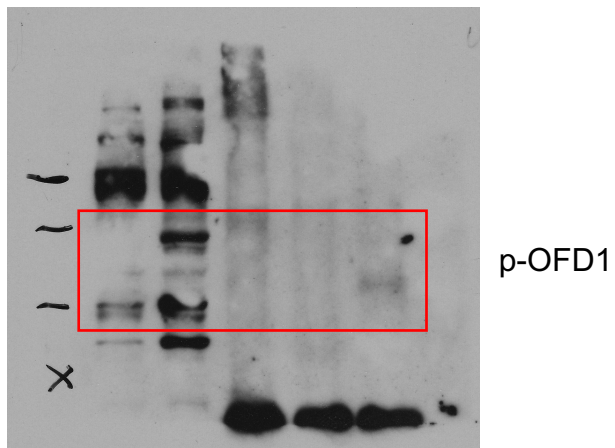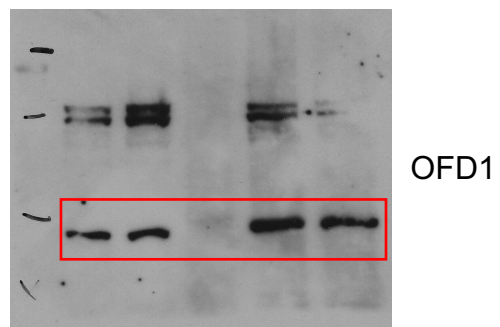

**Fig. EV3**

A

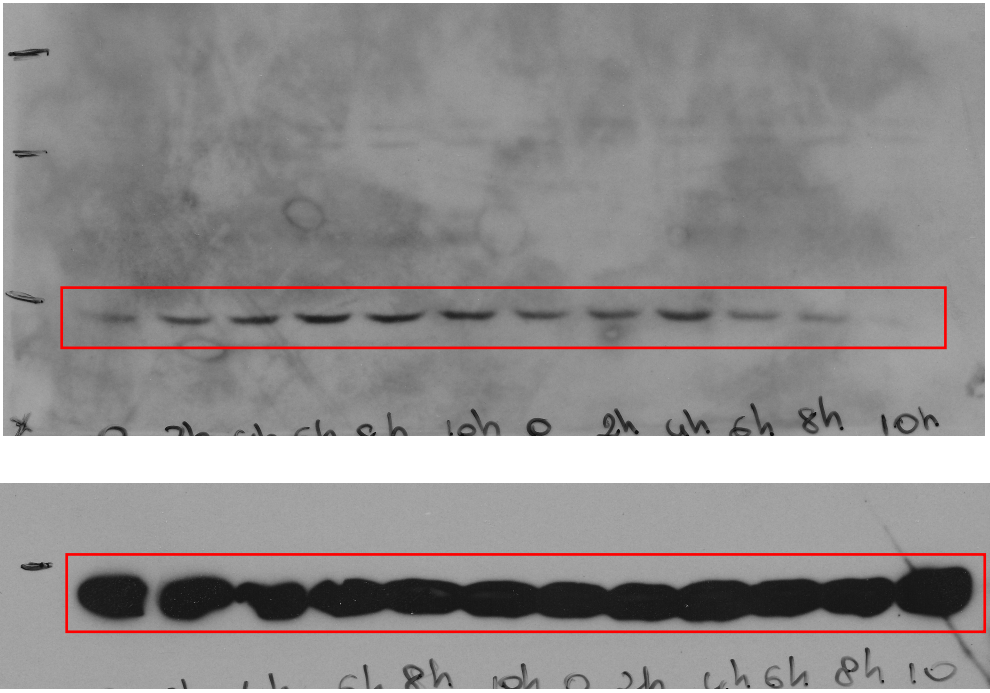

C

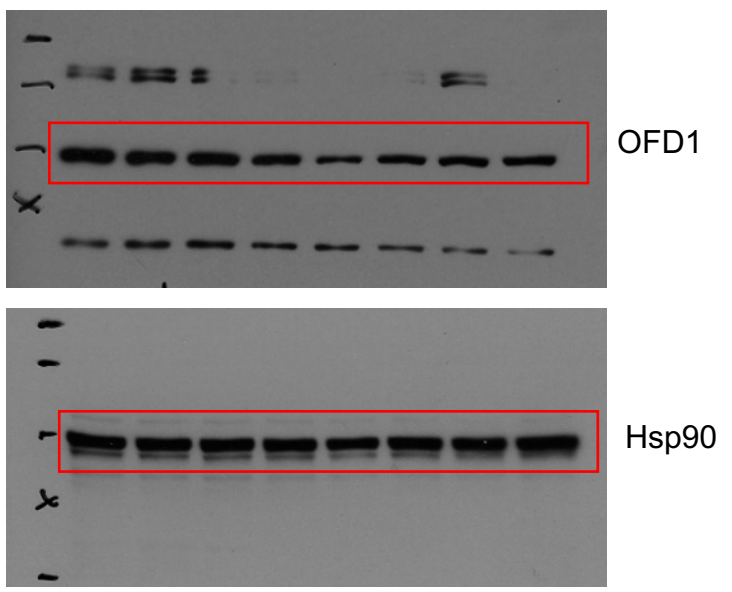

B

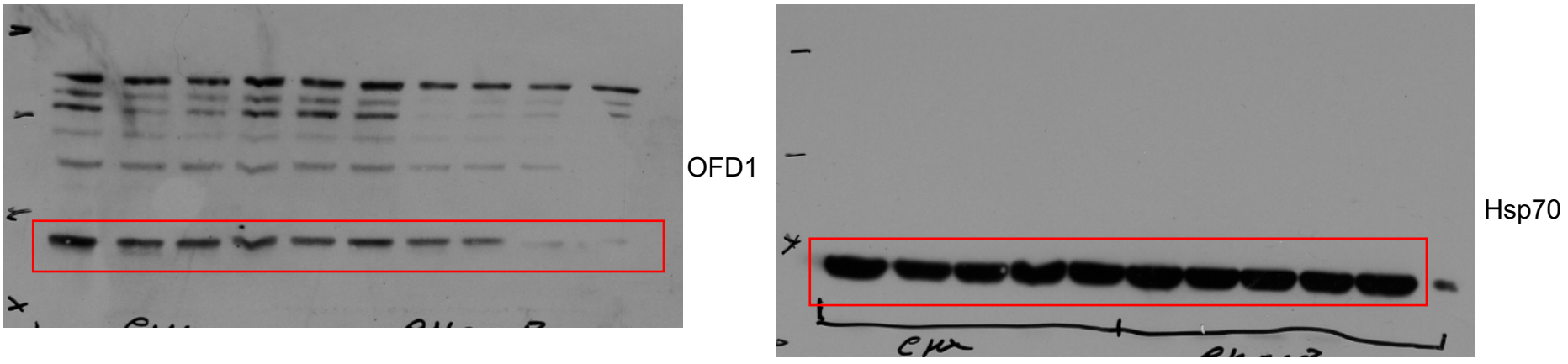

Fig. EV4

Supplement: Supplementary file 4 — Source Data for Expanded View [file EMBJ-40-e106503-s003.zip › EMBOJ-2020-106503_SourceDataForForExpandedView/EMBOJ-2020-106503_ourceDataForExpandedView.pdf]

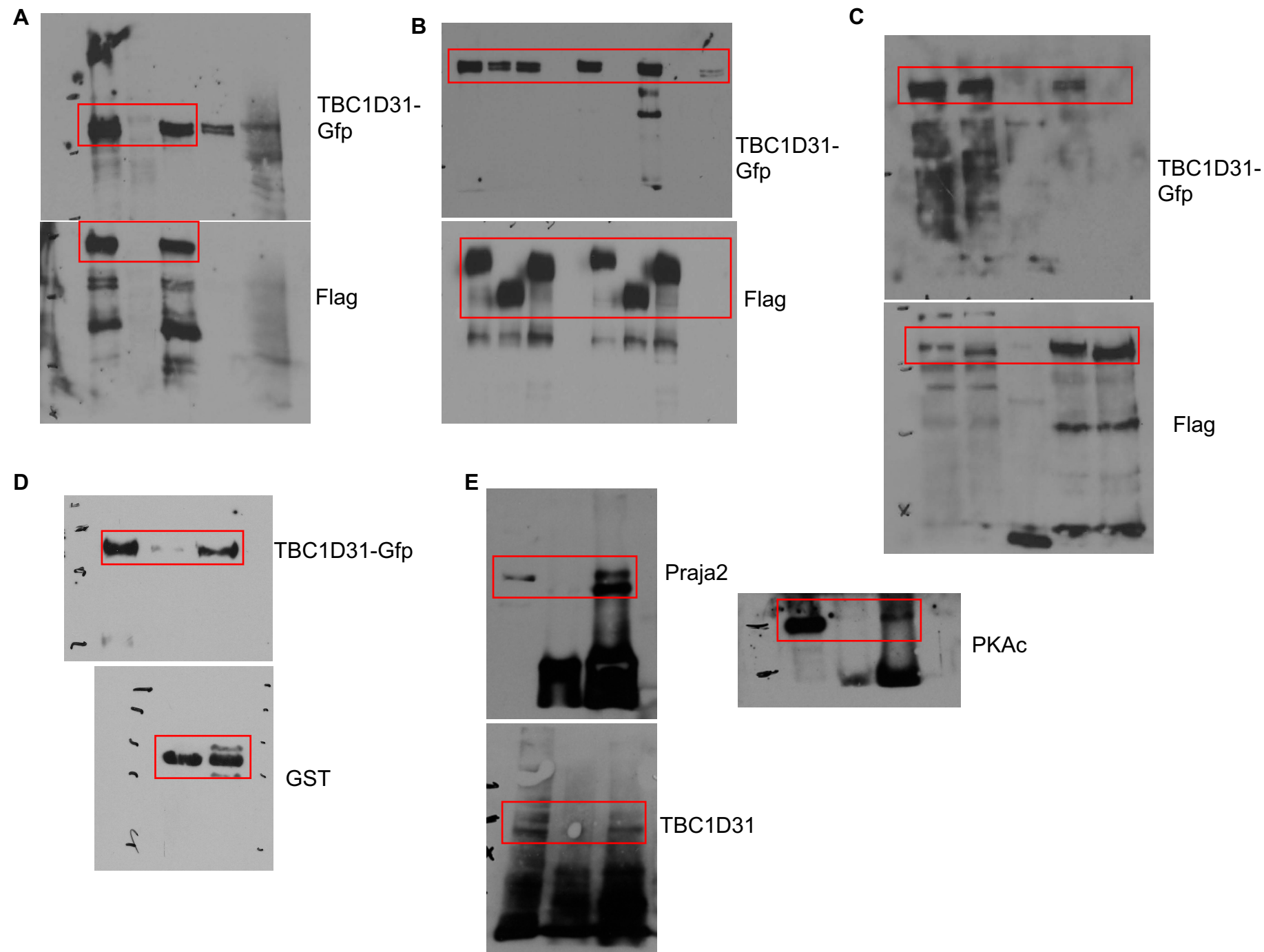

**Figure 1**

Supplement: Supplementary file 6 — Source Data for Figure 1 [file EMBJ-40-e106503-s006.pdf]

A

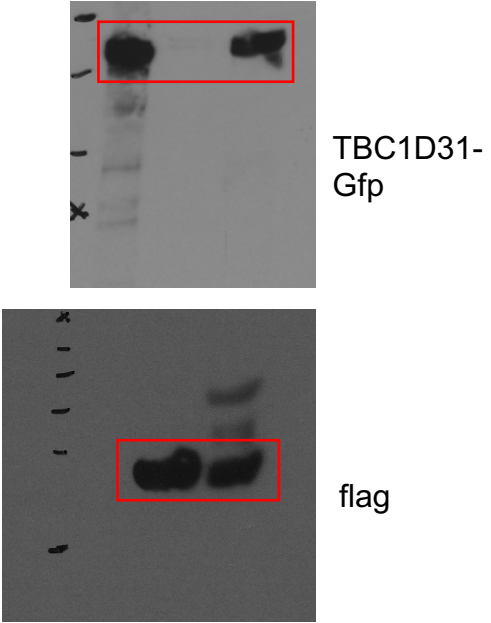

C

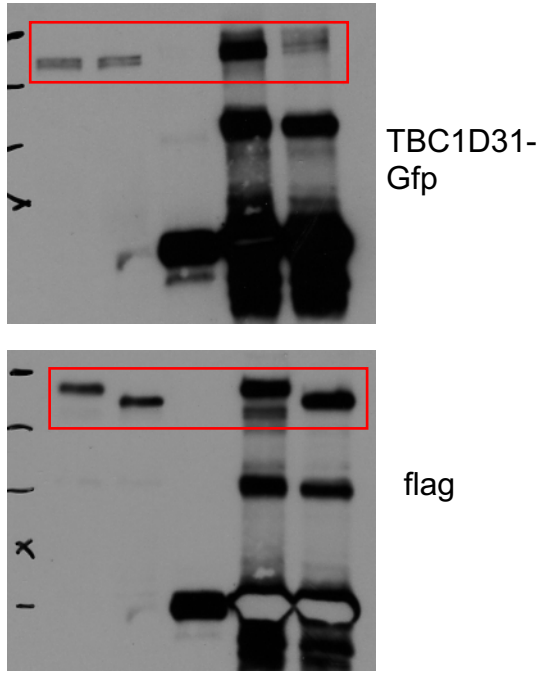

Figure 2

Supplement: Supplementary file 7 — Source Data for Figure 2 [file EMBJ-40-e106503-s001.pdf]

C

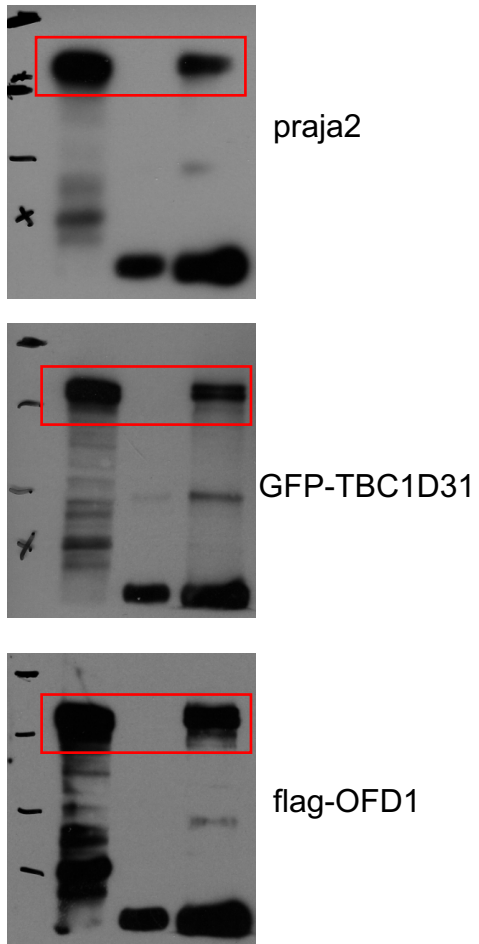

D

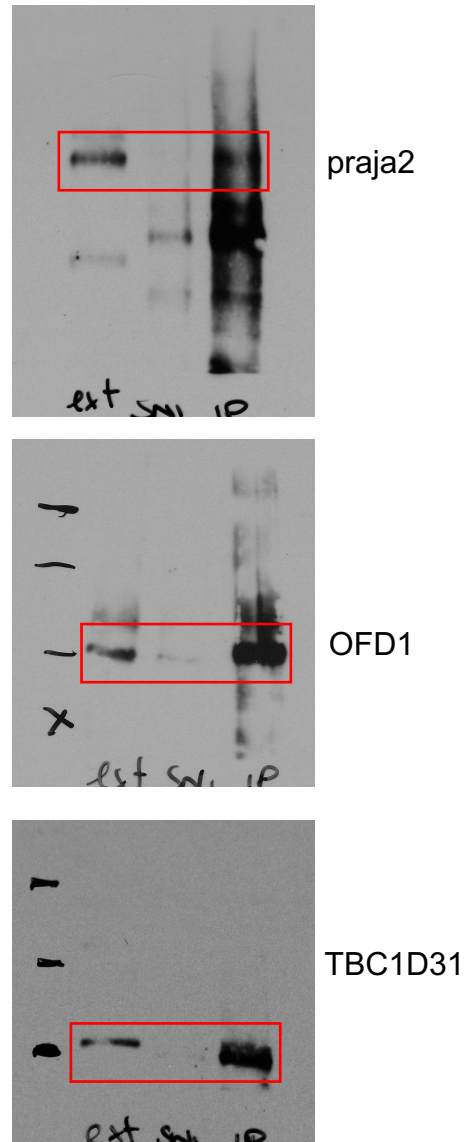

Figure 3

Supplement: Supplementary file 8 — Source Data for Figure 3 [file EMBJ-40-e106503-s005.pdf]

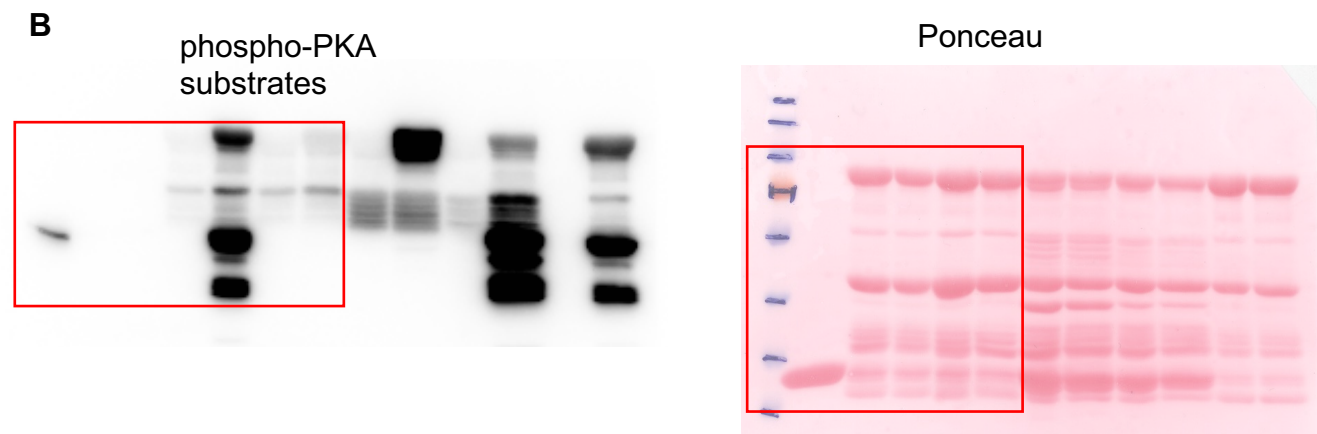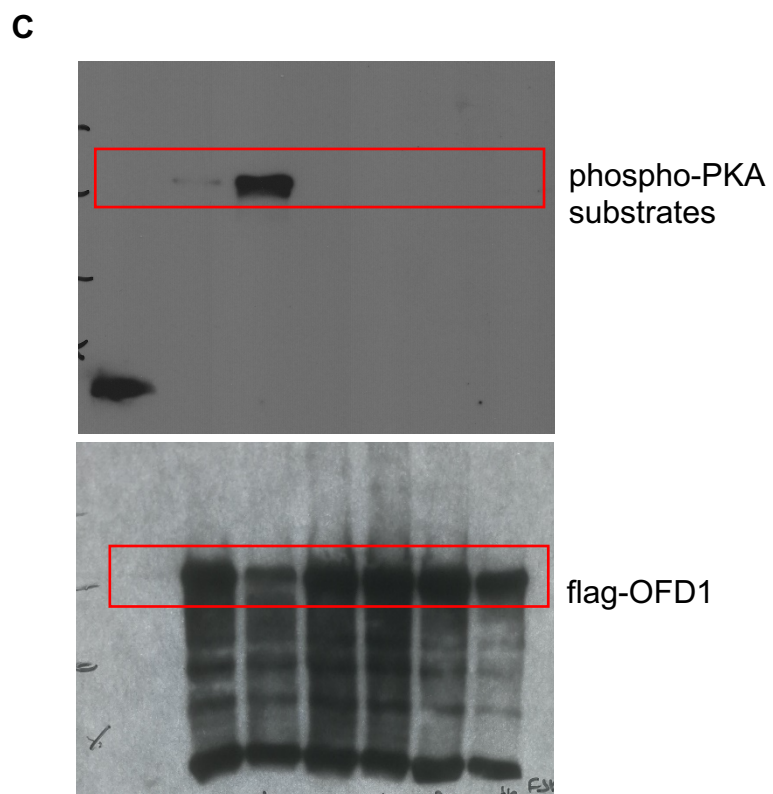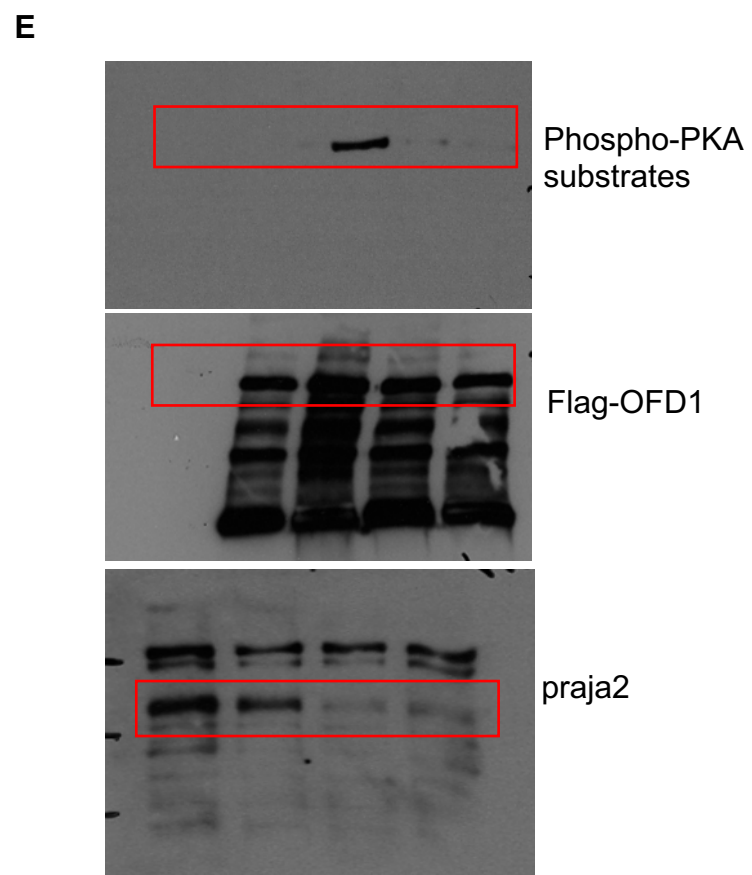

**Figure 4**

Supplement: Supplementary file 9 — Source Data for Figure 4 [file EMBJ-40-e106503-s009.zip › EMBOJ-2020-106503_SourceDataForFigure4[2].pdf]

**A**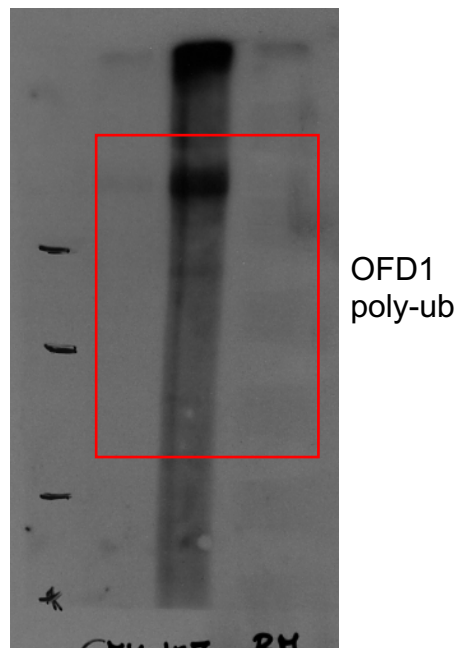**B**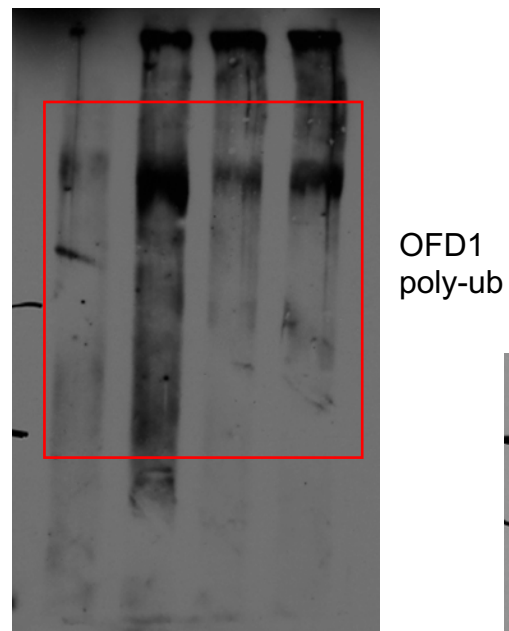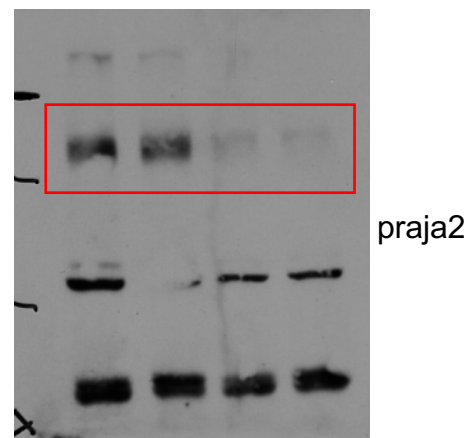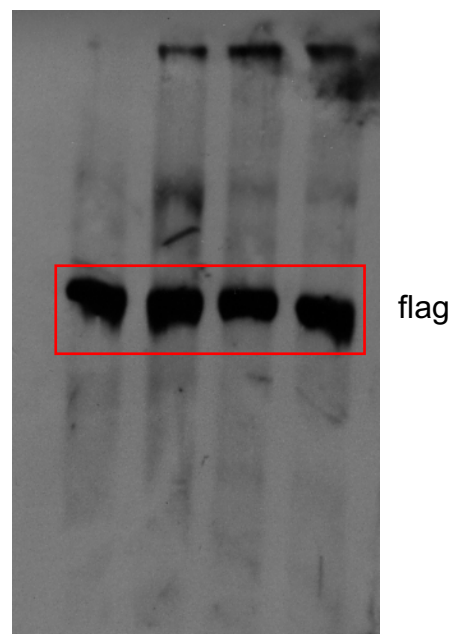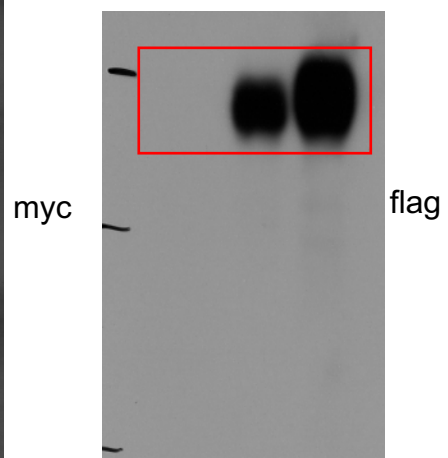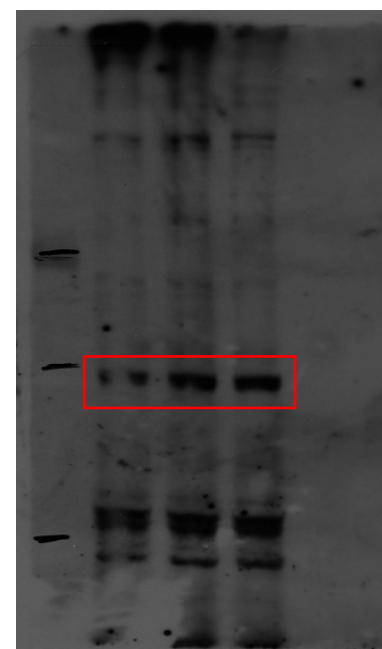**Figure 5**

Supplement: Supplementary file 10 — Source Data for Figure 5 [file EMBJ-40-e106503-s011.zip › EMBOJ-2020-106503_SourceDataForFigure5/EMBOJ-2020-106503_SourceDataForFigure5A-B.pdf]

C

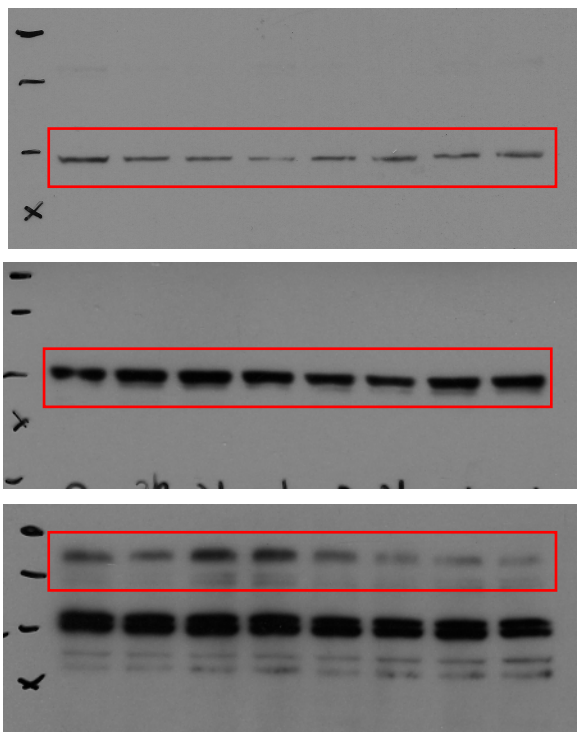

OFD1

Hsp90

praja2

E

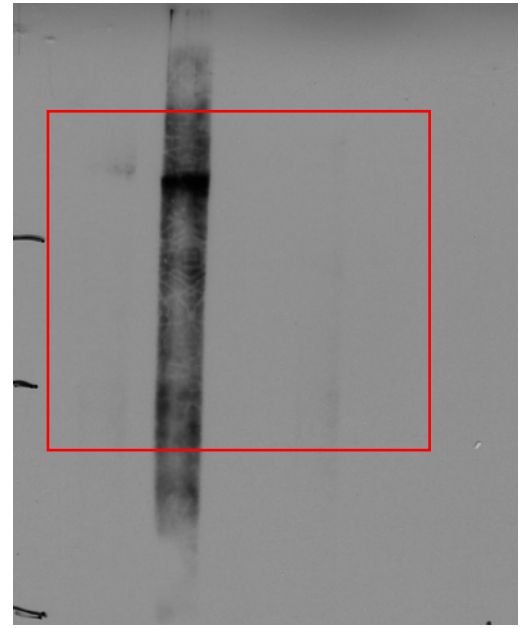

OFD1  
poly-ub

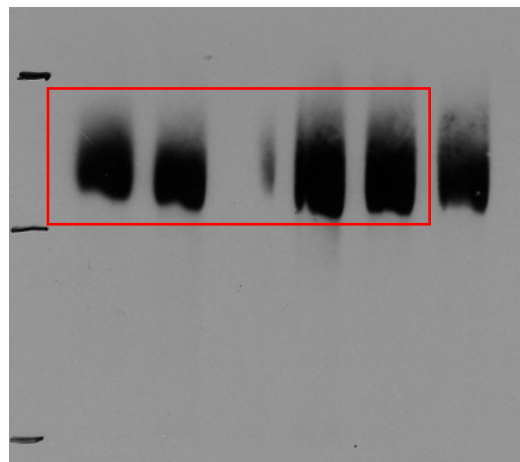

flag

Figure 5

Supplement: Supplementary file 10 — Source Data for Figure 5 [file EMBJ-40-e106503-s011.zip › EMBOJ-2020-106503_SourceDataForFigure5/EMBOJ-2020-106503_SourceDataForFigure5C-E.pdf]

**F**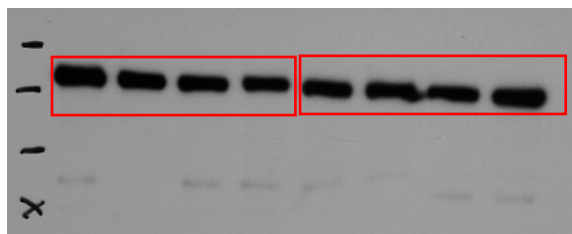

flag

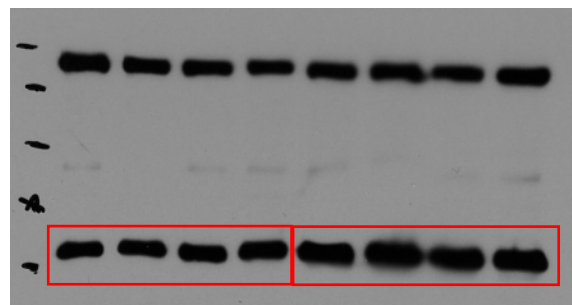 $\alpha$ -tub**H**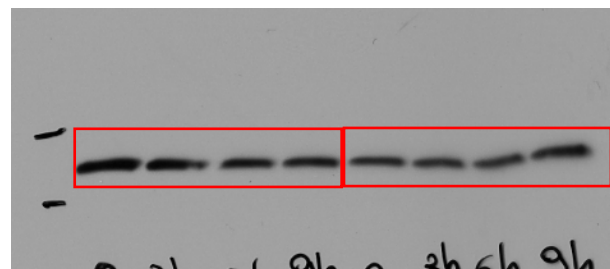

flag

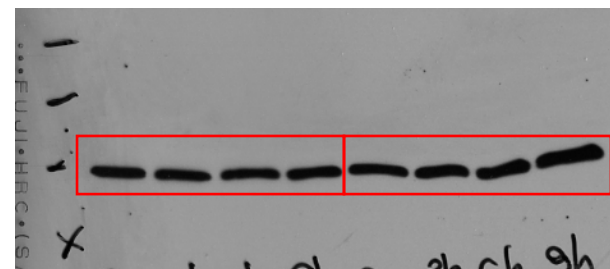

Hsp90

**Figure 5**

Supplement: Supplementary file 10 — Source Data for Figure 5 [file EMBJ-40-e106503-s011.zip › EMBOJ-2020-106503_SourceDataForFigure5/EMBOJ-2020-106503_SourceDataForFigure5F-H.pdf]
